# Drug use stigma, accidental pet poisonings, and veterinary care: results from a survey of pet owners in Vancouver, British Columbia

**DOI:** 10.3389/fvets.2025.1527196

**Published:** 2025-04-30

**Authors:** Chloe Chambers, Lexis H. Ly, Alexandra Protopopova

**Affiliations:** Animal Welfare Program, Faculty of Land and Food Systems, University of British Columbia, Vancouver, BC, Canada

**Keywords:** illicit drugs, animal welfare, companion animals, pet emergency, harm reduction

## Abstract

**Introduction:**

The effect of drug use on physical health, mental wellbeing, and quality of life can extend to family members, including children, and even companion animals. People who use drugs, a medically vulnerable population, face stigma and shaming when accessing healthcare services that engender mistrust and hinder future access. Yet, in an emergency where a pet has accidentally ingested drugs, there is no research on whether this stigma may prevent owners from seeking veterinary help. The objectives of this study were to describe actions taken by pet owners after accidental pet drug poisonings in Vancouver, British Columbia and understand how drug use-related stigma is associated with owners’ decision to seek veterinary care.

**Methods:**

We surveyed two populations of pet owners, a general population recruited online (*n* = 82) and a sample recruited in-person at two outreach services that assist low-income pet owners in the Downtown Eastside of Vancouver (*n* = 32). Participants who had not experienced a pet poisoning were asked about their actions and attitudes in a hypothetical drug poisoning event.

**Results:**

Within the general population sample, 64 (78%) responded based on a hypothetical scenario, and in this group, the concern that a veterinarian might remove their animal was associated with higher self-reported discrimination in three domains: general discrimination, discrimination accessing non-health services, and discrimination accessing health-related services. A higher perceived experience of discrimination within each category was also associated with a greater concern that veterinary staff might report them to the authorities or social services, as well as an increased expectation of hiding information from a veterinarian, including information about what drug the animal ingested and how the animal was exposed. Those who had experienced a poisoning emergency were asked about their actions and encounters in the most recent poisoning. More than half sought veterinary assistance. Among those that did not, some reasons owners avoided veterinary care included confidence in their ability to treat the animal at home (*n* = 5), or fear of discrimination (*n* = 1) or punishment (*n* = 1).

**Discussion:**

Our findings suggest that drug use-related stigma may contribute to a hesitancy to seek veterinary care or fully disclose information in an accidental pet poisoning.

## Introduction

The effects of drugs intended for human consumption on pets are increasingly discussed in articles, case studies, and other research on common pet toxins (1–3). After the legalization of marijuana in Canada, veterinarians anecdotally reported receiving more poisoning cases (4, 5). A survey of veterinarians in Canada and the United States found that most veterinarians did not report a change in the number of cannabis-induced toxicoses after 2018, but of those that did, almost all reported seeing an increase in the number (6). Two studies found an increase in the number of marijuana poisoning calls to the American Society for the Prevention of Cruelty to Animals (ASPCA) Poison Control Center following regulatory changes: medical marijuana licensing and state-level punishment for possession (7, 8). For canine opioid poisoning calls, a strong positive association was found between the county-level rate of opioid prescription and the probability of a poisoning call to the Poison Control Center (9). However, the authors were hesitant to suggest a causal relationship between drug use patterns and increased poisoning risk, as factors like decreased state punishment for possession of drugs or greater awareness of animal drug toxicoses by both owners and veterinarians may have also contributed to an increase in reports (6, 7). Veterinarians acknowledge that the illicit nature of many of these drugs may impact owner willingness to provide complete or accurate histories about how their animal may have ingested the substance (10, 11), highlighting a need to understand how stigma, discrimination, or perceived risk of judgment may impact owner willingness to seek veterinary care.

Stigma describes a set of negative attitudes toward people with a particular characteristic that has been deemed unacceptable by the social majority and can be seen in the forms of discrimination, judgment, stereotyping, marginalization, and other forms of social rejection (12, 13). Drug use, a behavior that is often perceived as socially undesirable, can be highly stigmatizing. In a Canadian survey of people who inject drugs, 88.7% reported experiences of discrimination for their drug use (14). In healthcare settings, negative attitudes held by physicians and care staff toward people who use drugs (PWUD) can affect the quality of care and treatment patients receive, including failure to respect patient dignity, provide prompt care, and provide basic amenities (15, 16). These negative experiences have been linked with an avoidance of healthcare by PWUD for various medical needs, not just drug harm-related, or may force patients to adopt strategies to minimize the harm caused by stigma, including not disclosing drug use, downplaying pain, seeking care elsewhere, or avoiding care altogether (15, 17). The consequences of stigma and healthcare provider attitudes on the health care received by PWUD have been established, but never examined in the context of veterinary care.

Drug use may intersect with other vulnerabilities, such as homelessness and poverty. Although the majority of drug toxicity deaths in British Columbia (BC) since 2018 have occurred in private residences (18, 19), vulnerably-housed individuals experience disproportionate harms and health risks associated with drug use (20–22). Among vulnerably-housed populations, pet ownership has been found to provide a sense of responsibility and purpose that can encourage owners to seek help with substance use issues or avoid situations where they might be separated from their animal, such as activities that could lead to arrest (23). However, homelessness and poverty present additional barriers to accessing various services, including both healthcare and veterinary care. LaVallee and colleagues (24) identified cost, physical accessibility, veterinarian-client relationships, cultural and language barriers, and a lack of education as the five most prominent barriers to veterinary care among low-income and vulnerably-housed pet owners. The intersection of these vulnerabilities with drug use-related discrimination may increase potential harm to pets and owners by acting as an additional barrier to veterinary care. Understanding how stigma interacts with barriers to veterinary care is valuable in overcoming issues of mistrust and a lack of communication between veterinarians and owners, and crucial in protecting the human-animal bond and companion animal welfare.

In our study, pet owners who use drugs, or had a recent history of drug use, were surveyed to understand circumstances surrounding hypothetical or actual instances of accidental pet drug poisonings. The first objective was to describe the nature of pet poisonings in Vancouver, including how pet owners handled, or would handle, such an emergency. The second objective was to determine if drug use-related stigma experienced by these pet owners was associated with increased fear or avoidance when seeking veterinary care. We predicted that drug use-related stigma would be linked with a stronger reluctance to seek veterinary care out of fear of stigmatization at the veterinary clinic, as well as a desire to avoid potential judgment, discrimination, or punitive action.

## Materials and methods

### Positionality statement

The research questions developed for the present study were the result of conversations with clients who were concerned about pet poisoning emergencies. AP is a scientist within the field of behavior analysis, animal behavior and welfare, and has an interest in animal sheltering and companion animal support programs. LL’s academic background is in animal welfare, and she has volunteered and collaborated with animal sheltering and human-animal support organizations. CC’s academic background is also in animal welfare, and she has worked and volunteered with organizations that serve several different vulnerable populations. The research team approaches research questions within animal welfare from a One Welfare perspective, with an understanding that animal issues are situated within larger societal contexts. The research team’s academic background warrants reflection on the angle from which we approached this topic, given our animal welfare focus and how it may influence the way we collected, analyzed, and reported data. None of the members of the research team have any lived experience with either drug use-related stigma or housing vulnerability. We gathered input from veterinary professionals who worked closely with vulnerably-housed pet owners, but we recognize that we did not gather input from individuals with lived experience, apart from informal conversations.

### Setting and participants

In 2016, the province of BC declared a public health emergency in response to the unprecedented number of toxic drug-related deaths, and has implemented several evidence-based initiatives to reduce the harms of the overdose crisis, including supervised consumption, overdose prevention, and drug checking services (25). A three-year pilot project to decriminalize the possession of small quantities (less than 2.5 g) of illicit drugs was introduced in January of 2023 with the hope of decreasing stigma surrounding drug use and encouraging access to health and social services (26) but was modified to recriminalize drugs in public spaces as of May 7^th^, 2024 (27).

We recruited 114 pet owners from the Greater Vancouver area in the province of British Columbia, Canada who were over the age of 18 and self-identified as someone who uses drugs. The survey was distributed to two populations using different collection methods: an online survey and an in-person survey. Both samples were convenience samples of different populations. Sampling was not done to assess generalizability, but rather to describe the experiences of pet owners.

The online survey aimed to capture data from a general population of pet owners in Vancouver. Recruitment for the online survey was conducted using social media posts, emails to pet service providers, and flyers posted in several high pet owner traffic areas, such as veterinary hospitals. The survey was available online via anonymous link or a QR code from March 6th to June 14th, 2023.

The second group of participants was recruited in-person at two outreach programs that serve low-income pet owners in the Downtown Eastside (DTES) neighborhood of Vancouver. This sub-population was of particular interest, as the DTES is characterized by a large population of people experiencing intersecting vulnerabilities, such as homelessness, financial hardship, and mental health challenges, including disproportionately high rates of drug use and drug use-related harm (28). Recruitment at the first location, Charlie’s Pet Food Bank, run by the BC Society for the Prevention of Cruelty to Animals (BC SPCA), occurred once a week for 12 weeks during the food bank’s operating hours (10 am-12 pm every Thursday). Recruitment at the second location, a veterinary fair hosted by Community Veterinary Outreach, was one day only from 11 am to 2 pm on June 3rd, 2023. The survey was administered in-person using iPad devices, as it was expected this population may have limited cellphone ownership or access to stable internet connection (29). Some participants reported having difficulty operating the iPad or had a visual impairment that prevented them from reading the questions. In these situations, the survey was conducted verbally, where the researcher read out the questions and options, selecting the indicated answer. Participants were not required to answer all the questions and could choose to leave the survey at any time. Online participants could enter their email in a draw for a $25CAD gift card, and all in-person participants were given a $10CAD gift card upon completion of the survey.

### Survey

Responses were collected through an online, anonymous survey hosted by Qualtrics survey software. The full survey for online participants can be found in a publicly available data repository (see Data availability statement). The in-person survey had two fewer questions: there was no reCAPTCHA for Internet bot detection and no email entry for the gift card draw, which was a separate survey linked to the original to maintain anonymity. The body of the survey consisted of 74 questions, including multiple choice, short answer, select all, and Likert-style, taking 15–20 minutes to complete. The first section verified participation criteria, asked demographic questions, and questions about the participant’s pet ownership. The second section asked about participant drug use habits in the last six months, including frequency, type of drug, delivery method, location of use, and whether other people in their household used drugs. Two Likert-style questions assessed respondents’ experience of stigma relating to their drug use, including discrimination from various groups, and barriers they face when accessing several services, like employment services (1 = Strongly disagree, 5 = Strongly agree). These questions were adapted from discrimination scale questions in Ahern and colleagues’ survey on illicit drug use stigma (30). A third Likert question asked about participant experience of discrimination within healthcare settings. Respondents could indicate how frequently they experience discrimination when seeking healthcare, as well as how often they might adopt avoidant strategies when seeking healthcare (1 = Never, 5 = Always) (15, 17).

The third section was headed by a question that divided participants into two streams: pet owners who had never experienced an accidental pet drug poisoning and those who had. Participants in the first stream were asked to imagine a hypothetical scenario, while participants in the second stream were asked to remember the most recent (or only) time this emergency happened to them. Five Likert-style questions established owner confidence, actions, and experiences in either a hypothetical or real pet poisoning emergency, with an additional Likert question within the hypothetical survey stream assessing owner concern that a poisoning might occur. These concerns were adapted from Ahern and colleagues’ (30) questionnaire but changed to reflect differences between human healthcare and veterinary care. Finally, participants had the opportunity to select from a list of suggestions what might make a veterinary visit safer and non-stigmatizing or could add their own suggestions. The survey also included open-ended questions at the end of each section for participants to elaborate on their experience or express anything they felt was necessary for clarification, but they were not included in the present analysis. The survey was piloted with members of the University of British Columbia Animal Welfare Program for clarity, accessibility, and duration.

### Data and analysis

Despite the Qualtrics survey software measures to prevent the inclusion of bot responses, we noticed several low-quality responses from the online survey. Several rounds of data cleaning were conducted, removing 762 responses. The criteria can be found in the Supplementary material. The data were analyzed using R version 4.3.0 (31). Multiple choice and select all demographic results and drug use characteristics are presented as counts and proportions for online and in-person survey data, with an additional combined total and proportions. Annual income and number of pets are reported as means and ranked questions as mean rank. Likert responses were converted to numeric values for the calculation of means, which were either reported descriptively or used in tests of correlation. Statistical analysis was performed only on questions within the online hypothetical survey stream (n = 64) to investigate relationships between drug use stigma and access to veterinary care using a large enough sample size. We tested the correlation of the four to five statements within each of the stigma-related Likert questions using Spearman’s correlation, then calculated a “stigma score” per respondent for each of the three questions that averaged the responses to statements within a question. The relationship was examined between these stigma scores and the Likert scores for seven potential concerns seeking veterinary care in a hypothetical poisoning, as well as three potential concerns sharing information with a veterinarian, using Spearman’s correlation. Similarly, we examined the relationship between the stigma scores and the type of veterinary or emergency service pet owners would access in a hypothetical poisoning. The sample from the in-person survey was too small to conduct any statistical analyses for comparison to the online population, but the data are described to highlight the findings from this population.

Data from the online and in-person surveys are presented and discussed separately in the Results and Discussion sections. As was expected given the distinct populations from which we sampled, our results indicated substantial demographic differences between the two survey methods (see details in Results, Table 1). Our decision to explore the in-person sub-population separately was also based on the expectation that individual- and community-level factors may be inextricable from our examination of pet drug poisoning events (32–34). Conclusions about the combined data may not reflect unique experiences and challenges faced by individuals facing one or more barriers, such as homelessness. To reflect that the observed differences are likely due to social vulnerability factors rather than a result of the data collection format, we will henceforth use the terms ‘general sample’ for the online convenience sample of general Vancouver residents and ‘outreach program clients’ for participants recruited and surveyed in-person at one of the two pet service outreach programs.

**Table 1:** Sociodemographic characteristics of survey participants.

Characteristic	General population sample (*n* = 82)	Outreach program clients (*n* = 32)	Combined(*n* = 114)
	*n (%)*	*n (%)*	*n (%)*
Gender identity	*n* = 81	*n* = 32	*n* = 113
Female	36 (44.4)	17 (53.1)	53 (46.9)
Male	41 (50.6)	12 (37.5)	53 (46.9)
Non-binary	3 (3.7)	2 (6.3)	5 (4.4)
Two-spirit	1 (1.2)	1 (3.1)	2 (1.8)
Age	*n* = 75	*n* = 30	*n* = 105
18–24	10 (13.3)	0 (0)	10 (9.5)
25–34	40 (53.3)	4 (13.3)	44 (41.9)
35–44	15 (20.0)	9 (30.0)	24 (22.9)
45–54	7 (9.3)	6 (20.0)	13 (12.4)
55–64	2 (2.7)	11 (36.7)	13 (12.4)
65+	1 (1.3)	0 (0)	1 (1.0)
Racial or Ethnic Background*	*n* = 82	*n* = 32	*n* = 114
White or European descent	42 (51.2)	19 (59.4)	61 (53.5)
Black	16 (19.5)	0 (0)	16 (14.0)
Hispanic or Latin American	13 (15.9)	0 (0)	13 (11.4)
Middle Eastern	8 (19.8)	1 (3.1)	9 (7.9)
East Asian	7 (8.5)	1 (3.1)	8 (7.0)
Southeast Asian	3 (3.7)	0 (0)	3 (2.6)
South Asian	0 (0)	0 (0)	0 (0)
First Nations or Indigenous	2 (2.4)	11 (34.4)	13 (11.4)
Another racial or ethnic background	1 (1.2)	3 (9.4)	4 (3.5)
Employment Status	*n* = 82	*n* = 32	*n* = 114
Full-time employment	48 (58.5)	1 (3.1)	49 (43.0)
Part-time employment	15 (18.3)	10 (31.3)	25 (21.9)
Financial aid/benefits	10 (12.2)	13 (40.6)	23 (20.2)
Piecework/contract work	5 (6.1)	2 (6.3)	7 (6.1)
Student	4 (4.9)	0 (0)	4 (3.5)
Retired	2 (2.4)	1 (3.1)	3 (2.6)
Unemployed	7 (8.5)	9 (28.1)	16 (14.0)
Prefer not to say	1 (1.2)	2 (6.3)	3 (2.6)
Housing Status	*n* = 82	*n* = 32	*n* = 114
House	14 (17.1)	0 (0)	14 (12.3)
Apartment/condo	11 (13.4)	0 (0)	11 (9.6)
Rented apartment/condo	26 (31.7)	17 (53.1)	43 (37.7)
SRO	2 (2.4)	9 (28.1)	11 (9.6)
Shelter	1 (1.2)	3 (9.4)	4 (3.5)
Staying with someone else (e.g. friend)	28 (34.1)	0 (0)	28 (24.6)
Unhoused	0 (0)	2 (6.3)	2 (1.8)
Trailer	0 (0)	1 (3.1)	1 (0.9)
Neighbourhood	*n* = 76	*n* = 28	*n* = 104
Downtown	22 (28.9)	21 (75.0)	43 (41.3)
West Side	21 (27.6)	0 (0)	21 (20.2)
Central Vancouver	16 (21.1)	5 (17.6)	21 (20.2)
East Vancouver	9 (11.8)	1 (3.6)	10 (9.6)
Outside of Metro Vancouver	8 (10.5)	1 (3.6)	9 (8.6)
Mean annual income (SD)	*n* = 65	*n* = 25	
	75, 108 (106, 893)	15, 315 (9,091)	

Sample sizes are reflected in the table on the first line of each variable. An asterisk (*) denotes a “select all” question.

## Results

The cleaned sample included 82 responses from the general population survey and 32 responses from the outreach program client survey. General sample respondents had an average of 1.7 (± 1.0) pets, with 57 respondents (69.5%) having at least one dog, 38 (46.3%) having at least one cat, 10 (12.2%) having at least one small mammal, and 4 (4.9%) having another species of pet. Outreach program clients had an average of 1.6 (±0.9) pets, with 23 (71.9%) having at least one dog, 15 (46.9%) having at least one cat, and 2 (6.2%) having at least one small mammal. Table 1 provides a summary of the sociodemographic characteristics of participants.

Because the potential effects of drug toxicosis in a companion animal depend on the type, composition, and quantity, participants were asked several questions about their drug use to get a view of potential poisoning risks, which are summarized in Table 2.

**Table 2:** Drug use characteristics of survey participants.

Characteristic	General population sample (*n* = 82)	Outreach program clients (*n* = 32)	Combined (*n* = 114)
	*n (%)*	*n (%)*	*n (%)*
Drug use frequency (past six months)	*n* = 82	*n* = 32	*n* = 114
I have used drugs once or twice	22 (26.8)	4 (12.5)	26 (22.8)
I have used drugs once or twice a month	14 (17.1)	4 (12.5)	18 (15.8)
I use drugs once a week	6 (7.3)	10 (31.3)	16 (14.0)
I use drugs multiple times a week	7 (8.5)	5 (15.6)	12 (10.5)
I use drugs every day	14 (17.1)	6 (18.8)	20 (17.5)
I use drugs multiple times a day	19 (23.2)	3 (9.4)	22 (19.3)
Drugs used*	*n* = 82	*n* = 32	*n* = 114
Cannabis	54 (65.9)	21 (65.6)	75 (65.8)
Hallucinogens	33 (40.2)	5 (15.6)	38 (33.3)
Cocaine/crack	17 (20.7)	11 (34.4)	28 (24.6)
Amphetamines	12 (14.6)	11 (34.4)	23 (20.2)
Depressants	11 (13.4)	2 (6.3)	13 (11.4)
Inhalants	10 (12.2)	7 (21.9)	17 (14.9)
Opioids	4 (4.9)	3 (9.4)	7 (6.1)
Steroids	1 (1.2)	1 (3.1)	2 (1.8)
Method of use*	*n* = 82	*n* = 32	*n* = 114
Smoking/inhalation	62 (75.6)	28 (87.5)	90 (78.9)
Oral consumption	32 (39.0)	9 (28.1)	41 (36.0)
Injection	26 (31.7)	5 (15.6)	31 (27.2)
Snorting	9 (11.0)	5 (15.6)	14 (12.3)
Do others in your household use drugs?	*n* = 63	*n* = 13	*n* = 76
Yes	33 (52.4)	7 (53.8)	40 (52.6)
No	30 (47.6)	6 (46.2)	36 (47.4)
Do you have measures in place to prevent others from accessing your drugs (e.g. children, pets)	*n* = 80	*n* = 31	*n* = 104
Yes	59 (73.8)	25 (80.6)	84 (75.7)
No	21 (26.3)	6 (19.4)	27 (24.3)

Sample sizes are reflected in the table on the first line of each variable. An asterisk (*) denotes a “select all” question.

### Description of pet poisoning emergencies in Vancouver, BC

Of all the survey participants (*n* = 114), 30 reported having experienced a pet drug poisoning: 18 (22.0%) from the general sample and 12 (37.5%) from the outreach program client group. Table 3 provides information about the frequency of these poisonings, and the type of poisoning respondents used to answer subsequent questions about the emergency.

**Table 3:** Accidental pet drug poisoning characteristics.

	General population sample (*n* = 18)	Outreach program clients (*n* = 12)	Combined (*n* = 30)
Characteristic	*n (%)*	*n (%)*	*n (%)*
How many times has this happened to you?			
Once	16 (88.9)	6 (50.0)	22 (73.3)
2-5 times	1 (5.6)	3 (25.0)	4 (13.3)
6-9 times	1 (5.6)	2 (16.7)	3 (10.0)
10+ times	0 (0)	1 (8.3)	1 (3.3)
What was the substance your pet ingested (most recent or only poisoning)?	*n* = 13	*n* = 7	*n* = 20
Cannabis	4 (30.8)	0 (0)	4 (20.0)
Cocaine	1 (7.7)	1 (14.3)	2 (10.0)
Hallucinogens	5 (38.5)	0 (0)	5 (25.0)
Inhalants	2 (15.4)	0 (0)	2 (10.0)
Opioids	1 (7.7)	4 (57.1)	5 (25.0)
Amphetamines	0 (0)	1 (14.3)	1 (5.0)
Other	0 (0)	1 (14.3)	1 (5.0)

Sample sizes are reflected in the table on the first line of each variable. The respondent who selected “Other” did not specify the drug.

Within the general sample that had experienced an accidental pet drug poisoning emergency (*n* = 18), 15 sought veterinary treatment, and either ended up having their animal treated there (*n* = 12) or treated them at home with advice from the veterinarian (*n* = 3). One person treated their animal at home without the advice of a veterinarian, and two participants did not seek treatment for their pet. Out of the outreach clients who responded to these questions (*n* = 11), two respondents took their animal to the veterinarian, with one receiving treatment there, and the other at home with veterinary advice. Six respondents treated their animal at home without the advice of a veterinarian, and three participants did not end up seeking treatment for their pet.

Respondents who took their animal to the veterinarian were asked to provide a rating of their overall experience with the veterinarian (1 = Extremely bad, 5 = Extremely good). Generally, respondents indicated that they had a more good than bad experience; the mean response for the general sample (*n* = 15) was 3.6 (±0.9) and 5.0 (±0.0) for outreach program clients (*n* = 2).

The participants who chose not to access veterinary treatment were able to select all the factors that contributed to this decision from a list of options or could select “Other” and describe further. From the general sample, all three participants who did not access veterinary care answered these questions. Two participants indicated they felt confident in their ability to handle the emergency, one person selected that a fear of criminal punishment played a role, and one selected that a fear of having their animal taken away played a role. One participant selected “Other,” clarifying that the veterinarian recommended no treatment.

Six participants from the outreach program population reported why they did not seek veterinary treatment. Three participants selected their confidence in their ability to handle the emergency and three selected the cost of the treatment. One cited fear of having their animal taken away, another was afraid of judgment and discrimination, and one was unable to physically access the treatment. Three participants chose to describe their reasons, selecting the “Other” option. Two indicated that the pet was getting better already, while the third said they did not know where to go to get assistance for a pet poisoning emergency.

### Owner responses to a hypothetical pet poisoning

The rest of the participants (*n* = 82) reported never having experienced a pet poisoning emergency. From the general sample, 64 (78.0%) answered based on a hypothetical occurrence of a pet drug poisoning, and from the outreach client sample, this number was 18 (56.3%). Participants generally agreed that they felt confident in handling this potential emergency (1 = Strongly disagree, 5 = Strongly agree); the general sample had a mean response of 3.7 (±1.0), while outreach program clients had a mean response of 3.8 (±1.4). Both groups also indicated that they would feel relatively confident in identifying symptoms of a pet drug poisoning [general sample = 3.7 (±0.9), outreach program clients = 4.2 (±1.3)].

For both populations, the highest ranked first action in a potential pet poisoning was to call a veterinary clinic or hospital. Table 4 shows the mean ranked order of possible first actions presented to the participants.

**Table 4:** Rank, mean rank, and standard deviation of different potential services that pet owners could access in a pet poisoning emergency.

	General population sample (*n* =62)		Outreach program clients (*n* =10)	
Rank	Service	Mean rank (SD)	Service	Mean rank (SD)
1	Call a vet clinic or hospital	4.0 (2.5)	Call a vet clinic or hospital	3.8 (2.9)
2	Emergency animal hospital	4.1 (2.4)	Call an animal poison line	3.8 (2.1)
3	Local vet clinic	4.2 (2.1)	Local vet clinic	4.4 (2.3)
4	Call an animal poison line	4.4 (2.5)	Treat at home	4.4 (2.3)
5	Call a human poison line	5.3 (2.4)	No action	5.0 (3.0)
6	Treat at home	5.3 (2.7)	Emergency animal hospital	5.2 (2.9)
7	Call a drug overdose line	5.8 (2.3)	Call a drug overdose line	5.7 (2.5)
8	No action	5.8 (2.8)	Community-based organization	5.7 (2.8)
9	Community-based organization	6.1 (2.5)	Call a human poison line	7.0 (1.5)

Participants were given a list of nine services and could rank which one they would be most likely to access.

### Drug use stigma and access to veterinary care in a hypothetical pet poisoning

While the intention was to examine drug use-related stigma and its effect on seeking veterinary care in both real and hypothetical poisoning emergencies in both demographic groups, the sample size of participants who had experienced a poisoning emergency (n = 30) was not large enough to provide desired statistical power, so the analysis was done in the general sample population who answered based on a hypothetical poisoning.

Participants were generally neutral when asked about their experiences of rejection or unfair treatment due to their drug use (1 = Strongly disagree, 5 = Strongly agree), their experiences with facing barriers (1 = Strongly disagree, 5 = Strongly agree), and how often they experienced discrimination accessing healthcare (1 = Never, 5 = Always). Participants reported comparable levels of agreement across questions regarding experiences rejection or unfair treatment from friends and family (3.0 ± 1.2), coworkers (3.1 ± 1.1), service employees (3.1 ± 1.2), hospital and healthcare staff (3.1 ± 1.1), and the general public (3.2 ± 1.2). Participants likewise had similar levels of agreement to questions on the extent to which they faced various barriers, including finding housing (3.0 ± 1.1), accessing social services (3.1 ± 1.1), accessing healthcare (3.1 ± 1.2), and finding employment (3.4 ± 1.2). Finally, participants responded consistently when asked how often they experienced discrimination accessing healthcare for drug use-related concerns (2.6 ± 1.1), put off accessing healthcare (2.8 ± 1.1), hid information about drug use (2.9 ± 1.1), sought alternatives to a clinic or hospital (2.7 ± 1.3), felt that their concerns are not taken seriously (3.2 ± 1.2), or felt fearful they would be reported to an authority (3.0 ± 1.3). Among respondents in the general sample that had not experienced a pet poisoning (*n* = 64), responses to the statements within each of these three questions were moderately to highly correlated with each other (Table 5). Respondents’ average stigma scores for each of the domains were therefore used for analysis.

**Table 5:** Range, mean, and standard deviation of Spearman correlation coefficients.

Domain of stigma	Range	Mean (SD)
Discrimination	0.48 – 0.76	0.70 (0.17)
Access to services	0.29 – 0.53	0.59 (0.27)
Healthcare	0.29 – 0.74	0.56 (0.23)

Two of the seven statements of concern about seeking veterinary care were moderately positively correlated with the average score across all three domains of stigma: discrimination, barriers to services, and healthcare-specific discrimination. Higher stigma was related to higher agreement with increased concern that veterinary staff might take away the owner’s animal (discrimination: *r_s_* = 0.51, *p* < 0.001, services: *r_s_* = 0.54, *p* < 0.001, healthcare: *r_s_* = 0.57, *p* < 0.001), and concern that veterinary staff might report them to the authorities or social services (discrimination: *r_s_* = 0.48, *p* < 0.001, services: *r_s_* = 0.58, *p* < 0.001, healthcare: *r_s_* = 0.46, *p* < 0.001). The concern that the veterinary staff might not take the emergency seriously was also moderately positively correlated with the increased average stigma score for barriers to services (*r_s_* = 0.51, *p* < 0.001) and healthcare-specific discrimination (*r_s_* = 0.40, *p* = 0.001). For a full summary of the remaining correlations, see Supplementary material. Respondent stigma scores for all three domains were all positively correlated with pet owner expectations to hide information from a veterinarian when seeking care in an accidental poisoning, with the strongest correlations being between both experiencing barriers to services and discrimination in healthcare settings, and an expectation to hide what drug the animal may have consumed (Table 6).

**Table 6:** Spearman correlation coefficients and *p*-values between willingness to disclose information to a veterinarian and average stigma scores for the three assessed domains (*n* = 64).

Domain of stigma	Discrimination		Access to services		Healthcare	
	*r_s_*	*p*	*r_s_*	*p*	*r_s_*	*p*
Statement						
You would expect yourself to hide information from veterinary staff about the nature of the substance the animal had consumed.	0.388	0.002	0.535	<0.001	0.540	<0.001
You would expect yourself to hide information from veterinary staff about how the animal could have gotten a hold of the substance.	0.317	0.011	0.491	<0.001	0.473	<0.001
You would expect to need to justify yourself to the veterinary staff upon revealing the nature of the substance.	0.328	0.008	0.410	<0.001	0.416	<0.001

Finally, we looked at whether experience of stigma was related to the rank of actions taken in a pet poisoning (Table 4). A higher healthcare stigma score was related to a lower mean rank (value closer to 1) for the decision to “wait/monitor symptoms” (*r_s_* = −0.28, *p* = 0.03).

## Discussion

### Descriptive findings

The first objective of our study was to describe accidental pet drug poisonings in the city of Vancouver, BC, and owner responses to these emergencies, given increasing concern from both veterinarians and pet owners over accidental pet drug poisoning incidents (4, 5). In our study, 26% of all survey respondents had experienced a pet poisoning, of which 27% had experienced more than one. While this sample is not representative of the overall prevalence due to convenience sampling, it does highlight a potential animal welfare concern. Over three quarters of our survey participants confirmed they had some kind of measures in place to prevent children or pets from accessing their drugs, and on average, owners expressed confidence in their abilities to identify and handle a pet poisoning.

Both general sample and outreach program clients ranked veterinary- or animal-related services within the top three actions they would take in a hypothetical emergency, but it is notable that the lowest mean ranks for both the general sample and outreach program clients were not close to one, and showed variability, indicating that there was no strong agreement among respondents toward a particular first action. Ultimately, in real poisoning emergencies, 83.3% of the general sample sought some kind of veterinary care or advice, while 16.7% of outreach program clients participants sought veterinary care, despite the proportion of pet drug poisoning emergencies being higher in the outreach group (22.0% versus 37.5%), which may reflect a difference between these two populations.

We observed differences in demographics and descriptive data, including mean annual income, housing status, neighborhood, and drug use characteristics that could influence both attitudes toward and actions in an accidental pet poisoning. Half of the outreach program program clients cited cost as the reason for not taking their animal to the veterinarian, which is consistent with previous findings that cost is one of the biggest barriers to accessible veterinary care (24, 35). Our study results suggest that people who do not seek veterinary care may seek other sources of information or rely on their personal knowledge of handling a drug overdose. However, drug effects in animals differ from those in humans, and veterinary treatment is always strongly recommended to address harmful symptoms (11). A lack of owner education and knowledge has been identified as a barrier to regular veterinary appointments among underserved communities, one that is heightened when owners face language, cultural, or cost barriers to care (24). Veterinary and animal welfare organizations that serve low-income pet owners could develop or share resources that provide accessible information to help owners understand pet poisonings and decide on cost-effective treatment options in a drug poisoning emergency. While many resources are available on the internet that provide information on symptom identification and veterinary treatment recommendations (36–39), for populations that do not have access to consistent internet connection or devices with which they can conduct internet searches, such as the Downtown Eastside (29), non-digital resources or educational material may be essential.

In our study, we also noticed differences between the general sample and outreach program clients regarding the type of drug ingested by the pet in the most recent poisoning event. Within the general sample, the majority of drug poisonings were related to cannabis or hallucinogens, while the majority of poisonings within the outreach program sample were opioid-related. Results from the Canadian Alcohol and Drug Use Monitoring Survey revealed that the proportion of people who used magic mushrooms in Vancouver and Victoria was over seven times higher in a “recreational drug use” group compared to a “high-risk” population of adults experiencing housing vulnerability, but whose use of heroin was 13 times lower (40). It may be that differences in drug use between people who are and are not experiencing homelessness explain this pattern in our data, but could also reflect survey selection or reporting bias (41, 42), or differences in comfort disclosing information about the type of drug consumed by the animal, which we found to be linked to drug use-related stigma. We also noted that the number of opioid-related poisonings for the outreach program population exceeded the number of respondents who reported using opioids, which suggests pets are potentially being exposed to opioids from various sources. Additional research that employs more comprehensive sampling methods to capture a larger, more representative population, or that explores the impact of environmental exposure, would be invaluable to further investigate these patterns and develop tailored resources to prevent accidental poisoning.

### Drug use stigma and access to veterinary care

The second aim of this study was to understand how drug use stigma might influence an owner’s decision to seek veterinary care in a pet poisoning. Our analysis of participants responding to a hypothetical drug poisoning event suggests that people who experience higher levels of drug use-related stigma, both in their everyday lives and when seeking services, including healthcare services, may have greater concerns seeking veterinary care.

A higher stigma score was associated with an expectation to hide information about the details of the poisoning from veterinarians, which aligns with existing literature on both accidental pet poisonings and behaviors to avoid harm in the healthcare system. In human healthcare contexts, marginalized groups and individuals, particularly those with past experiences of discrimination, are reluctant to disclose information to healthcare professionals (17, 43, 44). Veterinarians have also reported that owners can require prompting or reassurance before sharing information about a drug poisoning to a veterinarian, particularly when it is an illicit drug, making it challenging to provide treatment and can lead to unnecessary euthanasia (10, 11). Veterinarians could benefit from adopting harm reduction-based strategies used by healthcare providers to encourage transparent discussions about substance use, such as emphasizing the importance of knowing substance use history to make accurate diagnoses, the use of respectful language, or mindfulness about attitude and body language (45, 46). It is unclear, however, whether this reluctance to disclose details to a veterinarian revealed in our study indicates a protective strategy against discrimination, or whether it may be linked to fear of potential consequences, such as the removal of their animal.

We found a moderately correlated positive relationship between self-reported experiences of stigma related to drug use and concerns of having one’s pet taken away, as well as being reported to an authority when seeking veterinary care for an accidental pet drug poisoning. Several qualitative studies involving parents, predominantly mothers, who use drugs found that the fear their children may be seized and placed in protective services may be a significant driving factors behind a reluctance to seek drug use-related help (47–50). However, while veterinarians often have both professional and personal ethical commitments to report instances of animal cruelty, the authority to remove an animal from an owner lies with the local animal welfare enforcement bodies (51, 52). Mandated reporting plays a critical role in protecting welfare and rights of vulnerable groups, such as animals or children. However, it has more recently been examined in a critical light, with new understandings of racism, poverty, and other factors for health professionals to consider in the reporting process, and the growing concern that it may be used a tool of oppression against marginalized individuals (53–55). Research suggests that veterinarians tend to report instances of animal cruelty less than they feel is necessary due to fear of losing clients, breaking client trust, and an uncertainty to whom they should report the incident (56, 57). There is sparse discussion on the impact of mandated reporting by veterinarians on pet owners in the literature, particularly if they are vulnerable or marginalized, thus the need for future research in this area is two-pronged. First, research should seek to describe veterinarian perspectives and actions in the case of accidental pet drug poisonings, and understand a potential disconnect between pet owner perception of veterinarian jurisdiction to report animal cruelty, and actual veterinarian actions in similar drug poisoning cases. Second, it may be important to determine the sensitivity of veterinarian reporting of animal cruelty and whether similar patterns of bias exist in veterinary-mandated reporting, as they do in human services.

Overall, participants cited stigma-related factors, such as a fear of being judged, fear of criminal punishment, or fear of having their animal taken away as contributing to their decision not to seek veterinary care, but also barriers such as cost or accessibility. Several of these pet owners face multiple simultaneous barriers to veterinary care that make it difficult to extricate drug use-related stigma from all the interacting factors that impede access to veterinary care in general. A greater sample would be necessary to fully understand the effect of a participant’s perception of stigmatization on the type of care accessed or concerns seeking veterinary care, while controlling for factors, such as participant income or drug use characteristics, which may have influenced their decision-making in a hypothetical poisoning scenario.

### Limitations

The small sample size restricted the analysis of differences between the general sample and outreach program populations. For example, we did not assess the relationship between drug use stigma and a fear of stigmatization when seeking veterinary care among the outreach program clients, the population that saw a higher proportion of pet poisonings and a lower proportion of owners who sought veterinary care. Importantly, additional research conducted on this topic should aim to recruit a larger sample of individuals with actual experience of accessing services for accidental poisonings, particularly to understand how the vulnerabilities that intersect with drug use, such as poverty or homelessness, might also intersect with barriers to veterinary care in pet drug poisoning cases.

There may have been key differences between the populations that were not accounted for in the survey, such as varying openness to talking about their experiences with drug use and accidental drug poisoning. Within the field of substance use, there are concerns about the reliability and accuracy of self-reported data because of the perceived negative consequences to reporting truthfully (58). However, because of the positive rapport that existed between the low-cost pet care services and their clients, and the non-judgmental environment of these community outreach initiatives, it may be that outreach program participants felt more comfortable answering the survey questions truthfully. In the instances where the survey was conducted verbally for some participants recruited in-person, the research team noticed that participants would share additional information when answering multiple choice, select all, or Likert questions, sometimes to explain a response or discuss nuances the question failed to capture. Anonymous surveys allow participants to more freely express their thoughts or feelings toward a sensitive issue, such as drug use (59), but even with the efforts of the research team to create a survey that was non-stigmatizing in its language and questions, it may be that respondents still felt ashamed or fearful if talking about a past pet poisoning emergency. Survey validation methods, such as cognitive interviewing to reduce response errors and that pilots using a small sample of the intended respondents would perhaps allow for the creation of a more precise tool (60, 61). Additionally, a future project that employs in-depth qualitative methods, such as a semi-structured or unstructured interview, could be crucial not only to better understand how stigma interacts with other factors in deciding whether to seek veterinary care, particularly when participants may have multiple intersecting barriers, but also to provide greater opportunity for owners to explain their decision-making processes.

## Conclusion

We found that accidental pet drug poisoning experiences varied among pet owners who use drugs in Vancouver, BC. Pet owners were generally confident in their abilities to identify and act in pet poisoning emergencies and have trust in veterinarians to treat their pet in such an emergency. However, the experience of discrimination and drug use-related stigma by pet owners was related to hesitancy to seek veterinary care, and in some cases, was related to complete avoidance. Our results also suggest that veterinary-owner communication in poisoning emergencies may be an area that requires improvement. Future research could seek to further understand veterinarian perspectives and actions in the case of accidental pet drug poisonings, including ethical concerns and confidence handling such emergencies. Veterinarians can be encouraged to discuss their commitment and their responsibility as veterinarians with the client to help encourage full transparency in pet drug poisonings. For vulnerable populations, barriers to seeking veterinary care are equally obstructive in the context of accidental drug poisoning emergencies and should be considered explicitly in the development of resources or veterinary practice recommendations.

## Data Availability

The raw data, cleaned data, R code and survey are all available in the following public-access GitHub repository, Chambers C Manuscript Data: https://github.com/cchambers-ubc/Chambers-C-Manuscript-Data.

## Supplementary material

Supplementary Table 1 Chambers et al. Table of criteria used for survey data cleaning.

Supplementary Table 2 Chambers et al. Full table of Spearman correlation coefficients and p-values between concerns seeking veterinary treatment and average stigma scores for the three assessed domains.
