# Supplementary material for: Drug use stigma, accidental pet poisonings, and veterinary care: results from a survey of pet owners in Vancouver, British Columbia
Source: Front Vet Sci. 2025 Apr 30;12:1527196. doi: 10.3389/fvets.2025.1527196 (PMC12075221; doi:10.3389/fvets.2025.1527196)
Supplement: Supplementary Table 1 — Chambers et al. Table of criteria used for survey data cleaning. [file Table_1.docx]

**Supplementary Table 1:** Exclusion criteria used for cleaning survey responses. Criteria are listed in the order they were applied to the whole dataset. A first round of filtering was conducted using R studio. A secondary round of cleaning was conducted in Excel for short answer responses. Responses would be flagged, then if another exclusion criterion was met, the response would be taken out.

| *Exclusion criteria* | *Description* |
| --- | --- |
| Survey completion | Surveys that had not been completed (as assessed by Qualtrics software) were excluded. Surveys where questions had been skipped were still included. |
| Participant criteria | Surveys where the respondent did not meet both participant criteria were excluded: pet ownership, drug use. |
| reCAPTCHA score | Surveys with a reCAPTCHA score of < 0.5 were excluded. This value was guided by the Qualtrics fraud detection support that indicates a reCAPTCHA value of <0.5 is likely a bot respondent (42) |
| Attention check | Surveys were participants failed to respond the correct answer (Somewhat Disagree) to the attention check question were excluded |
| Non-English answers | Surveys that included short-answer responses in languages other than English were excluded. Filtering occurred in Excel; researcher discretion was used. |
| Duplicate short answer responses | Surveys where answers to the short answer questions (23, 31, 48, 66, 67.1) were verbatim or close-to-verbatim duplicates were removed. Filtering occurred in Excel; researcher discretion was used. |
| Unrelated answer | Surveys where short answer responses were not related to the question were removed. Filtering occurred in Excel; researcher discretion was used. |
| AI/search engine-generated answer | Surveys where short answer responses resembled too closely an AI or search engine generated answer were removed. Filtering occurred in Excel. Responses would be copied and pasted into a search engine to examine similarities; researcher discretion was used. |
| Answer consistency | Some questions in the survey had mutually exclusive answers (40) e.g. “have prior relationship [with the veterinarian]” and “DO NOT have prior relationship [with the veterinarian]” or answers that should have remained consistent (95, 102). Surveys where there was an inconsistency in answers were removed. Filtering occurred in Excel; researcher discretion was used. |
