# Supplementary material for: Drug use stigma, accidental pet poisonings, and veterinary care: results from a survey of pet owners in Vancouver, British Columbia
Source: Front Vet Sci. 2025 Apr 30;12:1527196. doi: 10.3389/fvets.2025.1527196 (PMC12075221; doi:10.3389/fvets.2025.1527196)
Supplement: Supplementary Table 2 — Chambers et al. Full table of Spearman correlation coefficients and p-values between concerns seeking veterinary treatment and average stigma scores for the three assessed domains. [file Table_2.docx]

**Supplementary Table 2.** Spearman correlation coefficients and p-values between concerns seeking veterinary treatment and average stigma scores for the three assessed domains.

| Domain of stigma | Discrimination | | Access to services | | Healthcare | |
| --- | --- | --- | --- | --- | --- | --- |
|  | *r_s_* | *p* | *r_s_* | *p* | *r_s_* | *p* |
|  |  |  |  |  |  |  |
| *Statement of concern* |  |  |  |  |  |  |
|  |  |  |  |  |  |  |
| I would be concerned that the veterinary staff might judge me for my drug use. | 0.268 | 0.032 | 0.300 | 0.016 | 0.176 | 0.165 |
| I would be concerned that the veterinary staff might not take my emergency seriously. | 0.349 | 0.005 | 0.511 | 0.000 | 0.406 | 0.001 |
| I would be concerned that the veterinary staff might deem me an unfit owner. | 0.353 | 0.004 | 0.262 | 0.036 | 0.324 | 0.009 |
| I would be concerned that the veterinary staff might accuse me of engaging in animal cruelty. | 0.301 | 0.016 | 0.321 | 0.010 | 0.350 | 0.005 |
| I would be concerned that the veterinary staff might take away my animal. | 0.509 | 0.000 | 0.537 | 0.000 | 0.565 | 0.000 |
| I would be concerned that the veterinary staff might report me to the authorities or social services. | 0.482 | 0.000 | 0.575 | 0.000 | 0.459 | 0.000 |
| I would be concerned that I would receive a lecture from veterinary staff about my drug use/other health habits. | 0.254 | 0.043 | 0.177 | 0.162 | 0.217 | 0.085 |
